# Transcriptomic analysis of three *Veillonella* spp. present in carious dentine and in the saliva of caries-free individuals

**DOI:** 10.3389/fcimb.2015.00025

**Published:** 2015-03-26

**Authors:** Thuy Do, Evelyn C. Sheehy, Tonnie Mulli, Francis Hughes, David Beighton

**Affiliations:** ^1^Department of Oral Biology, School of Dentistry, University of LeedsLeeds, UK; ^2^Department of Periodontology, Dental Institute, King's College LondonLondon, UK; ^3^Department of Periodontology, School of Dental Sciences, University of NairobiNairobi, Kenya

**Keywords:** *Veillonella*, caries, stress proteins, pH regulation, RNA-sequencing

## Abstract

*Veillonella* spp. are predominant bacteria found in all oral biofilms. In this study, a metatranscriptomic approach was used to investigate the gene expression levels of three oral *Veillonella* spp. (*V. parvula, V. dispar* and *V. atypica*) in whole stimulated saliva from caries-free volunteers and in carious lesions (*n* = 11 for each group). In the lesions the greatest proportion of reads were assigned to *V. parvula* and genes with the highest level of expression in carious samples were those coding for membrane transport systems. All three *Veillonella* spp. increased expression of genes involved in the catabolism of lactate and succinate, notably the alpha- and beta-subunits of L(+)-tartrate dehydratase (EC 4.2.1.32). There was also significantly increased expression of histidine biosynthesis pathway in *V. parvula*, suggesting higher intra-cellular levels of histidine that could provide intra-cellular buffering capacity and, therefore, assist survival in the acidic environment. Various other systems such as potassium uptake systems were also up regulated that may aid in the survival and proliferation of *V. parvula* in carious lesions.

## Introduction

*Veillonella* are obligate anaerobic Gram-negative small cocci isolated from the oral cavity and intestinal tract of humans and animals that gain energy from the utilization of short-chain organic acids, particularly lactate and succinate (Delwiche et al., 1985). The human *Veillonella* are *Veillonella parvula, V. atypica, V. dispar, V. montpellierensis, V. denticariosi*, and *V. rogosae* (Mays et al., 1982; Rogosa, 1984; Jumas-Bilak et al., 2004; Byun et al., 2007; Arif et al., 2008). The predominant *Veillonella* species on the tongue were *V. rogosae, V. atypical*, and *V. dispar* (Beighton et al., 2008; Mashima et al., 2011). *V. parvula* has often been detected as the predominant *Veillonella* species isolated from active occlusal carious lesions (Arif et al., 2008; Beighton et al., 2008). Based on these studies, each *Veillonella* species seems to occupy different intra-oral habitats with limited degree of overlap between species. With the pH of carious lesions reported to be below 5 (Hojo et al., 1994), the bacteria's ability to colonize and proliferate in such an environment necessitates them to exhibit a phenotype characterized by acid resistance. The objective of this study was to determine and compare the transcriptome of three of the predominant human oral *Veillonella* (*V. parvula, V. dispar*, and *V. atypica*) present in caries lesions and in the saliva of caries-free individuals.

Many bacterial genome sequence data are now publicly available, making it possible to exploit the opportunities offered by next generation sequencing (NGS) approaches to determine the *in vivo* expression of specific bacterial genes of individual species present in mixed-population biofilms. The short reads obtained from NGS can be aligned to bacterial genomes, enabling transcriptomic analysis of species without the need for species-specific protocols, as is necessary with the micro-array approach. The functional potential of the oral microbiome has been investigated using metagenomic approaches in which genomic DNA is extracted, sequenced and the resulting sequences annotated by comparison to extant complete and partial genome sequences (Belda-Ferre et al., 2012; Luo et al., 2012). To investigate gene expression, the metatranscriptome of an individual species within a natural biofilm may be determined using RNA sequencing (RNA-seq). The application of RNA-seq to the study of bacterial transcriptomes has been reviewed by Pinto et al. (2011) and McLean (2014). Several studies have also recently described the use of RNA-Seq as a tool to investigate the oral microbiome in health and disease (Duran-Pinedo et al., 2014; Jorth et al., 2014) as well as interrogate specific metabolic pathways in oral bacterial species *in vitro* (Zeng et al., 2013).

In this study, we adopted a metatranscriptomic approach to investigate the level of genes expressed by the three *Veillonella* in both active carious lesions and saliva of caries-free subjects, in order to observe metabolic activities occurring in their natural environment, which may give an insight into their intra-oral distribution.

## Materials and methods

### Samples collection and RNA isolation

Ethical approval was obtained for the collection of carious lesions (*n* = 11) and saliva (*n* = 11) samples. All subjects (*n* = 22) gave informed consent prior to collection of the clinical material. Extracted teeth with large occlusal soft, active carious lesions were obtained from patients attending dental clinics at Guy's Hospital dental surgery. The teeth were immediately placed in 5 ml RNAprotect® Bacteria Reagent (Qiagen) and transferred to the laboratory. The superficial biofilm was carefully removed and discarded. The infected soft dentine was collected using sterile excavators, and placed in 1 ml RNAprotect reagent, disaggregated, centrifuged (4°C at 10,000 × g) and the pellets stored at −80°C. Whole mouth wax-stimulated saliva samples, collected for 5 min, were obtained from caries-free volunteers who refrained from eating for at least 2 h prior to sampling. Immediately after collection, RNAprotect reagent was added to the saliva (1:1 v/v), the samples were centrifuged and the pellets stored at −80°C until further processing. Total RNA was extracted using the UltraClean® Microbial RNA isolation kit (MOBIO Laboratories, Inc.), including a DNase treatment step using the RNase-Free DNase Set (Qiagen) prior RNA elution.

### cDNA synthesis and library preparation for high-throughput sequencing

A minimum of 100 ng of total RNA was extracted from each clinical sample. The total RNA was processed using reagents provided in the Illumina® TruSeq™ RNA Sample Preparation Kit. Briefly, the RNA extracts were further purified, and fragmented. First and second strands cDNA were synthesized with Superscript II Reverse Transcriptase (Invitrogen). End repair was performed on the nucleic acid fragments, 3′ ends were adenylated and adapter indexes ligated. The processed cDNA were amplified and further purified, prior library validation with the Agilent DNA 1000 Bioanalyzer (Agilent Technologies) and dsDNA BR Qubit assays (Invitrogen). The resulting libraries were processed for cluster generation using the TruSeq paired end cluster kit v.2, Illumina Inc., and an equimolar amount of each library was run in a separate flowcell lane. Paired end sequencing was then carried out using a Genome Analyzer IIx Illumina platform to produce 76 bp reads.

### Data handling and gene expression analyses

A FASTQ file was obtained for each of the 22 cDNA libraries. Initial checks of the sequencing read base qualities were done via the local server provided by the Genomics facilities at Guy's Hospital Biomedical Research Centre, the data were then imported into the CLC Genomics Workbench software (CLC Bio, Qiagen). Within the CLC environment, adapter sequences were removed and for each sample file a short read mapping was performed simultaneously against 144 annotated oral bacterial genomes which were previously imported from various databases (the DNA Data Bank of Japan, NCBI, the Broad Institute and HOMD databases) (Supplementary File 5). The read mapping was carried out using the RNA-Seq analysis package default settings (mismatch cost: 2, insertion cost: 3, deletion cost: 3, length fraction: 0.8, and similarity fraction: 0.8; with the maximum number of hits for a read set to 1) within the CLC software, which employs the CLC Assembly Cell (CLC3) read mapper (http://www.clcbio.com/products/clc-assembly-cell/).

In this study, we are concerned with reads that mapped to 3 *Veillonella* strains: *V. parvula* DSM2008, *V. dispar* ATCC 17748, and *V. atypica* ACS 0049 V Sch6 only (Table 1). In order to facilitate comparison between these *Veillonella* strains, a RAST annotation (Rapid Annotation using Subsystem Technology) (Aziz et al., 2008) was carried out on their genomes and used with the other oral strains in the read mapping. All of the 22 sequence data files were processed for read mapping against the 144 oral genomes. Results were exported as excel files containing raw read counts determined for each of the genes from the 144 oral strains (total of 351,456 genes) (Supplementary File 1). In order to compare expression levels between the 22 biological samples, the raw count data were gathered into a single excel spreadsheet for normalization (Supplementary File 1). The read counts were scaled by determining the effective library size of each sample, using the estimateSizeFactors and counts accessor functions within the Bioconductor R package DESeq (Anders and Huber, 2010), which provided an output table displaying normalized expression values for each gene and for each of the 22 samples. Data corresponding to the 3 *Veillonella* strains were manually extracted from the spreadsheet and used separately for further analysis to infer on their gene expression levels in caries lesions and caries-free saliva samples. Median values were calculated for both caries and saliva sample groups (*n* = 11 each) (Supplementary File 2), which we called relative median expression (RME) values. The RME values of identical genes found in the 3 *Veillonella* strains were summed and ranked from highest to lowest values, to observe the most highly expressed *Veillonella* transcripts in caries and caries-free saliva samples (Supplementary File 2). The gene identities were obtained from the RAST annotations and was supplemented by BLAST searching within Uniprot (http://www.uniprot.org/), InterProScan (http://www.ebi.ac.uk/Tools/pfa/iprscan/) and PATRIC (http://patricbrc.org/) when necessary.

**Table 1:** **Characteristics of the 3 *Veillonella* strains selected in this study, with their relative proportions in caries and saliva metatranscriptomes (CDS refers to coding genes)**.

**Feature code**	***Veillonella* species**	**strain**	**Number of CDS (from RAST annotation)**	**Relative proportion (%) of transcripts in caries (*n* = 11) ±SD**	**Relative proportion (%) of transcripts in saliva (*n* = 11) ±SD**
HMPREF	*V. atypica*	ACS-049-V-Sch6	1840	0.91 ± 0.43	4.09 ± 3.47
VEIDISOL	*V. dispar*	ATCC 17748	1954	2.18 ± 1.13	7.08 ± 5.07
Vpar	*V. parvula*	DSM 2008	1904	16.62 ± 11.17	4.76 ± 7.21

The raw read count data from all 144 oral strains were also used to carry out differential gene expression analysis between both sample groups using the statistical software R package DESeq2 (Love et al., 2014) based on the negative binomial model. Differential expression analysis results for the 3 *Veillonella* strains were manually extracted from the total R result outputs into excel spreadsheets, and the largest negative and positive Log2 Fold Change values, with adjusted *p*-values (padj) < 10^−3^ were considered as significant.

The Supplementary File 1 contains the raw count input information used for the DESeq and DESeq2 analyses.

### Sequence data accession numbers

RNA-Seq sequencing data are available from the National Center for Biotechnology Information (NCBI) Sequence Read Archive; biosamples accession numbers for this study are SRS741215 and SRS752041.

## Results and discussion

### Analysis of read count and ecological considerations

Here we have determined gene expression levels by mapping reads to bacterial species which form part of the oral microbial populations. The total number of mapped reads ranged between 25,593,022 and 88,238,546 for the caries-free saliva samples and between 20,088,245 and 32,910,299 for the caries samples (Supplementary File 1). In the carious lesions, 16.62 ± 11.17 per cent, 2.18 ± 1.13 per cent and 0.91 ± 0.43 percent of the mapped reads were assigned to *V. parvula, V. dispar*, and *V. atypica*, respectively, compared with 4.76 ± 7.21, 7.08 ± 5.07, and 4.09 ± 3.47 in the saliva samples (all *p* < 0.05) (Table 1). The pattern of the distribution of reads mirrored the reported distribution of these three species based on cultivable bacterial studies (Arif et al., 2008; Beighton et al., 2008). Belda-Ferre et al. (2012) also reported *V. parvula* to be the most predominant species in biofilm infecting dentine, with 166 contigs (>500 bp) assigned to *V. parvula* from their metagenomic data.

The major environmental factors affecting the *Veillonella* strains in the carious lesions and in saliva are suspected to be the low pH and availability of organic acids (lactate and succinate) required for the generation of ATP. The acidic environment within carious lesions is unlikely to be homogenous despite lactic acid being the major organic acid present (Palmer et al., 2006), resulting in areas that might be more alkaline (i.e., pH > 6). Nevertheless, it should be expected that the concentration of organic acids in saliva is less than that of carious lesions, since subjects had refrained from eating for 2 h prior sample collection, hence organic acids and dietary components should have cleared from the mouth. Moreover, we should emphasize that the microbiota present in wax-stimulated saliva is likely to derive from the intra-oral mucosal surfaces and from the supra-gingival plaque, providing an average composition of intra-oral surfaces, but mostly of the tongue surface (Simon-Soro et al., 2013). These ecological aspects have been taken into account and explain the differences in metabolic activities occurring within the *Veillonella* species in both sample groups.

### Gene expression analysis

**Figure 1:**
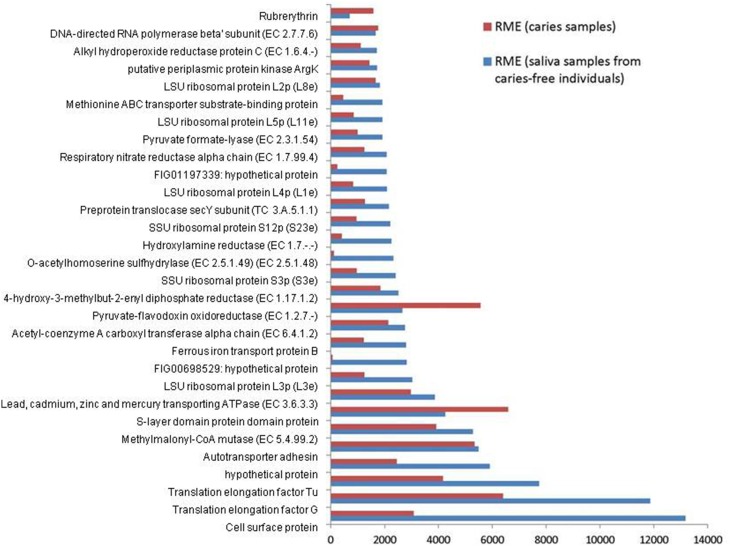
**Relative median expression (RME) levels in 3 *Veillonella* strains (*V. parvula* DSM2008, *V. dispar* ATCC 17748 and *V. atypica* ACS 0049 V Sch6)**. RME were calculated from the median values of normalized read counts in the caries (*n* = 11) and saliva (*n* = 11) samples. The 30 highest RME values were sorted in ascending order for the genes in saliva samples and are displayed with the RME values of corresponding genes in caries samples.

The combined expression level of the 3 *Veillonella* species was determined for each condition. The relative mean expression (RME) values for each identical gene product were added and the top 30 most highly expressed gene products in saliva were ranked. The corresponding values for the caries samples are also displayed together in Figure 1.

Overall, the 3 *Veillonella* species present in the caries and saliva samples display a similar profile of transcripts. *V. parvula* expressed more genes in the caries samples, whereas *V. dispar* expressed more genes in the saliva samples (Table 1, Supplementary File 2).

The most abundant transcripts were related to the production of cell surface proteins (RME = 13175), outer membrane synthesis (S-layer proteins, RME = 6592), translation elongation factors (G and Tu, RME = 11860 and 7731 respectively), transport systems (RME = 5481), ribosomal subunit proteins (protein biosynthesis, RME = 3028), and carbohydrate metabolism (particularly the glyoxylate and dicarboxylate metabolism, EC 4.2.1.32, EC 6.4.1.2, EC 1.1.1.37; RME = 2147, 2126, and 1461 respectively). These results are consistent with those described by Peterson et al. (2014) in plaque biofilm. Similarly Benítez-Páez et al. (2014) found evidence of overrepresentation of translation functions, together with high expressions of elongation factors Tu and G, emphasizing their importance and involvement in oral biofilm formation especially in early biofilms.

We also report high levels of transcripts encoding membrane transport proteins (cadmium-exporting ATPase, RME = 3861; autotransporter adhesin, RME = 5481; ABC tranporters, RME = 1998), as well as transcripts involved in oxidative stress protection (rubrerythrin, RME = 1577; and alkyl hydroperoxide reductase protein C, EC 1.6.4.-, RME = 1700), in both caries and saliva groups (Supplementary File 2). The overall similarity in transcription profiles in both sample groups suggest that the selected *Veillonella* species are actively expressing genes that are involved in cellular maintenance and survival within diverse environments.

### Differential expression analysis

Differential gene expression between the caries and saliva groups was investigated using the R package DESeq2 (Love et al., 2014).

**Figure 2:**
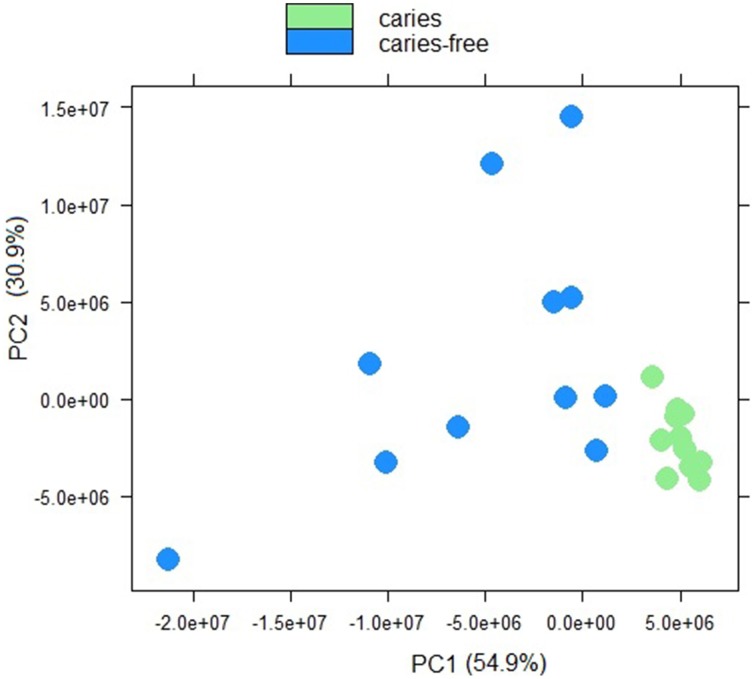
**Principal component analysis plot displaying sample-to-sample distances for caries and saliva samples**. The PCA plot is based on the differential expression analysis of 144 oral bacterial strains, carried out using the R package DEseq2 (Love et al., 2014).

**Figure 3:**
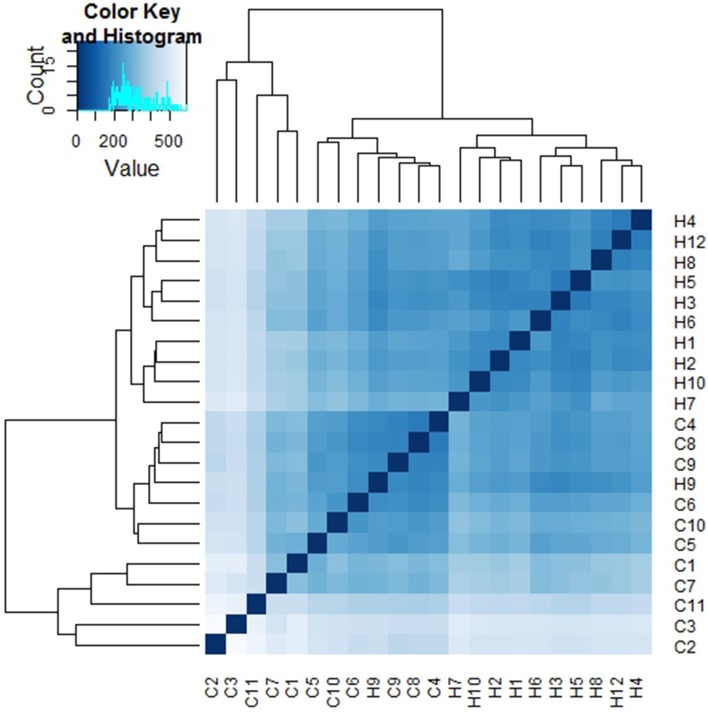
**Heatmap of Euclidian distances between samples (*n* = 22)**. The heatmap was constructed using the R package DESeq2 (Love et al., 2014), and is based on the differential expression analysis of 144 oral bacterial strains.

Sample to sample distances were calculated within the DESeq2 package. The principal components analysis and the heatmap of Euclidian distance between samples were based on the metatranscriptomic data mapped to 144 oral strains, and show caries and saliva samples to form distinct clusters. The PCA plot displays larger differences between saliva samples than between caries samples (Figure 2), suggesting that metabolic functions in the caries lesions are more conserved that in the caries-free samples. Likewise, the heatmap indicates the overall similarity between samples of the same group, with the exception of saliva sample number 9 (H9 in Figure 3), which seems to cluster with the caries samples, indicating that it shares similar functions found in caries.

Jorth et al. (2014) found more similar functional features in microbiota associated with disease compared to health-associated microbiota, even though great variations in the oral microbial composition were observed between and within patients. Other papers have described inter-patients variations in terms of bacterial profiles, and these seem to reduce in diversity when changing from healthy to a disease status (Munson et al., 2004; Preza et al., 2008). Our data suggest that in the caries lesions, metabolic functions in the 3 *Veillonella* species are more similar, than in caries-free saliva samples.

In order to identify the main functional differences between the caries lesions and saliva samples, output data from the DESeq2 analysis were sorted according to the log2 fold change values (Supplementary File 3). Since the transcriptomic data (*n* = 22) were analyzed with the caries-free vs. caries condition (used as the default DESeq2 condition setting), negative log2 fold change values, with corresponding Benjamini-Hochberg (BH) adjusted *p*-values (padj) < 10^−3^ considered as significant (Benjamini and Hochberg, 1995), indicate genes with the strongest down-regulation in saliva (or strongest up-regulation in caries). Conversely, the largest log2 fold change values, with corresponding significant BH padj < 10^−3^, indicate genes which are the most differentially expressed in saliva. Only the top 15 genes in both conditions are displayed in Table 2, and ranked according to the log2FoldChange values. A heatmap was also constructed within the DESeq2 package, and displays the top 30 differentially expressed genes across all 22 samples for the 3 *Veillonella* species (Supplementary File 4).

**Table 2:** **Up-regulated genes in the caries samples (top of table) and in the saliva samples (bottom of table)**.

**Feature ID**	**Gene product**	**base mean**	**log2Fold change**	**lfcSE**	**padj**
Vpar_1291	L(+)-tartrate dehydratase beta subunit (EC 4.2.1.32)	357.26	−8.90	0.72	2E-31
Vpar_1292	L(+)-tartrate dehydratase alpha subunit (EC 4.2.1.32)	646.86	−7.83	0.74	2E-22
VEIDISOL_00680	L(+)-tartrate dehydratase beta subunit (EC 4.2.1.32)	84.70	−6.60	0.81	1E-13
VEIDISOL_00681	Possible membrane transport protein	5.72	−6.28	1.09	2E-07
Vpar_0720	hypothetical protein	2.99	−6.17	1.32	4E-05
Vpar_1308	Ornithine carbamoyltransferase (EC 2.1.3.3)	41.52	−5.84	1.03	3E-07
Vpar_0455	Sulfur carrier protein ThiS	6.55	−5.73	1.18	2E-05
Vpar_1307	N-acetyl-gamma-glutamyl-phosphate reductase (EC 1.2.1.38)	36.32	−5.51	1.02	1E-06
Vpar_1306	Acetylglutamate kinase (EC 2.7.2.8)	28.84	−5.28	1.11	3E-05
Vpar_0164	FIG01197475: hypothetical protein	20.37	−5.28	1.04	7E-06
Vpar_1004	Alpha-aspartyl dipeptidase Peptidase E (EC 3.4.13.21)	18.93	−5.26	1.12	3E-05
Vpar_0330	FIG01197189: hypothetical protein	159.84	−5.00	0.80	2E-08
VEIDISOL_00679	L(+)-tartrate dehydratase alpha subunit (EC 4.2.1.32)	63.25	−4.96	0.94	3E-06
Vpar_1022	Putative ATP:guanido phosphotransferase (EC 2.7.3.-)	114.18	−4.74	0.92	5E-06
Vpar_1367	RND efflux system, outer membrane lipoprotein, NodT family	40.60	−4.63	0.86	1E-06
HMPREF9321_0616	Siroheme synthase / Precorrin-2 oxidase (EC 1.3.1.76)	6.02	6.78	1.66	3.8E-04
VEIDISOL_01296	Cold shock protein CspC	5.35	6.80	1.63	2.8E-04
HMPREF9321_1331	Ferrichrome transport ATP-binding protein FhuC (TC 3.A.1.14.3)	14.49	6.86	1.21	3.4E-07
HMPREF9321_0811	FIG002958: hypothetical protein	5.70	6.90	1.62	2.0E-04
HMPREF9321_0294	Small-conductance mechanosensitive channel	5.82	6.94	1.62	1.7E-04
HMPREF9321_1565	Exopolyphosphatase (EC 3.6.1.11)	5.67	6.94	1.61	1.6E-04
HMPREF9321_0879	Homocysteine S-methyltransferase (EC 2.1.1.10)	18.40	7.04	1.21	1.5E-07
HMPREF9321_1453	Molybdenum cofactor biosynthesis protein MoaB	6.44	7.10	1.60	9.3E-05
VEIDISOL_01157	Sodium-dependent transporter	7.78	7.28	1.59	5.8E-05
HMPREF9321_0702	FIG01197118: hypothetical protein	8.58	7.33	1.60	5.4E-05
HMPREF9321_0668	NAD(P)HX epimerase/NAD(P)HX dehydratase	8.15	7.34	1.59	4.4E-05
HMPREF9321_0246	Mobile element protein	11.60	7.74	1.56	1.1E-05
HMPREF9321_1665	binding-protein-dependent transport systems inner membrane component	13.47	7.88	1.56	7.5E-06
VEIDISOL_00207	Alkanesulfonates/ Sulfonate ABC transporter, ATP-binding protein	14.99	7.96	1.57	6.2E-06
HMPREF9321_1134	Oligopeptide ABC transporter, periplasmic oligopeptide-binding protein (TC 3.A.1.5.1)	17.60	8.24	1.53	1.6E-06

*Genes expressed with the strongest down-regulation in the saliva samples (or up-regulation in the caries lesions) and genes with the strongest up-regulation in saliva samples were determined using the R package DESeq2 (Love et al., 2014). The list of genes is ranked according to the Log2FoldChange values from the negative lowest values (strongest down-regulation in saliva) to the positive highest values (strongest up-regulation). The baseMean corresponds to the average of the normalized count values (divided by size factors), the log2FoldChange corresponds to the effect size estimate indicating the change in gene expression between both sample groups; lfcSE corresponds to the standard error of the log2FoldChange estimate, and padj corresponds to the Benjamini & Hochberg adjusted p-values*.

Genes that were differentially expressed in caries lesions (padj < 10^−3^) were those expressed by *V. parvula*, and were mainly involved in pyruvate metabolism, transferases and membrane transport systems (including the biosynthesis of efflux pump components, ABC transporter and sulfur carrier proteins) (Table 2), inferring a role of these functions in disease. Similar findings were reported by Benítez-Páez et al. (2014) who found that ABC transporters were significantly up-regulated in mature biofilms, with cell motility function associated with bacterial chemotaxis, whereas Duran-Pinedo et al. (2014) reported significant levels of ABC transporters in periodontitis samples that seem associated with high levels of expression of virulence factors. Other specific pathways associated with disease have also been reported. In the case of periodontitis, a significant enrichment in butyrate production was detected (Jorth et al., 2014), iron acquisition and membrane synthesis have also been described as important metabolic activities defining disease (Duran-Pinedo et al., 2014).

However, in our data all 3 *Veillonella* species (especially *V. parvula*) expressed genes involved in glyoxylate and dicarboxylate metabolism, and alanine aspartate and glutamate metabolism, in particular genes encoding the alpha- and beta-subunits of L(+)-tartrate dehydratase (EC 4.2.1.32). These are involved in the production of ATP through catabolism of lactate and succinate. Overall, the data suggest that all species responded to growth in the carious lesions by increasing the expression of many genes associated with the utilization of lactate and succinate with the consequent generation of ATP via the sodium ion-translocating methylmalonyl-CoA decarboxylase (Buckel, 2001). We also found significant up-regulation of genes encoding aspartate aminotransferases (Vpar_1105, Vpar_0075, HMPREF9321_0571, HMPREF9321_1684) in both caries and saliva samples (Supplementary File 3); these enzymes catalyze the reaction L-aspartate + 2-oxoglutarate into oxaloacetate + L-glutamate and may be an alternative method of entering intermediates into the lactate metabolic pathway, for producing ATP.

Genes involved in histidine metabolism were also up-regulated in caries by *V. parvula*, but not in the other 2 species (Supplementary File 3). Of particular importance is the up-regulation of ATP phosphoribosyltransferase (EC 2.4.2.17) which has a central role in histidine biosynthesis. Similar up-regulation was observed in *Corynebacterium glutamicum* and *Salmonella typhimurium*, as well as in *Lactobacillus casei*, in response to acid adaptation (20 min at pH 4.5) (Foster, 1995; Brockmann-Gretza and Kalinowski, 2006; Broadbent et al., 2010). It was suggested that the up-regulation of the histidine operon resulted in increased intra-cellular levels of His which may contribute to intracellular buffering capacity as the pK_a_ value of the imidazole groups of histidine and histidine-containing peptides is near 6.0 and these have been shown to contribute to intracellular buffering in vertebrate cells (Abe, 2000).

Additionally, a potassium uptake system in *V. parvula* (Vpar_1334 and Vpar_1335; KtrA and KtrB) was also significantly up-regulated in caries, but not by the other 2 species. K^+^ uptake in prokaryotes is essential for maintenance of cytoplasmic pH (Csonka and Epstein, 1996; Stumpe et al., 1996), this system may also assist in the survival of *V. parvula* in the acidic environment of the carious dentine. In *V. dispar* and *V. dispar*, these genes were significantly up-regulated in saliva, which may explain their lower ability to control their intracellular pH in the caries lesions. Clearly, *V. parvula* exhibits several distinct systems for intracellular pH control which do not appear to function as well in either *V. atypica* or *V. dispar*, and this may explain the ability of *V. parvula* to be better fitted to growth and proliferation in the acidic environment of carious lesions compared to the other two species.

Most of the differentially expressed genes in the saliva samples are those expressed by *V. atypica and V. dispar*, and encode for the oligopeptide, sulfonate transporter systems, and cysteine and methionine metabolism (EC 2.1.1.10). Others include genes involved in purine metabolism (EC 3.6.1.11), ferrichrome and other transport systems, molybdenum cofactor biosynthesis, as well as oxidoreductases which involve the use of NAD+ or NADP+ as acceptor in the chemical reaction leading to the formation of siroheme from uroporphyrinogen III (EC 1.3.1.76) (Table 2).

General stress response genes have also been identified in the carious lesions and saliva samples in all 3 *Veillonella* species (Supplementary File 3). Several genes encoding heat shock and chaperonin proteins were found up-regulated in caries (Vpar_1034, Vpar_1035, Vpar_0881), and others up-regulated in saliva (VEIDISOL_01212, HMPREF9321_0106, VEIDISOL_01142). Stress proteins such as chaperonin heat shock protein 33 (HMPREF9321_0536) and putative peroxide-responsive repressor PerR (HMPREF9321_0995) were up-regulated by *V. atypica* in the saliva samples (Supplementary File 3). In *V. parvula* and *V. dispar*, several genes associated with extracellular S-layer formation, were significantly up-regulated, which is a well characterized stress-associated response (Xiao et al., 2012).

## Conclusion

Recent reports of metagenomic and metatranscriptomic analyses of oral samples are providing extensive information on the microbial populations and functions characterizing health and disease. These studies have also confirmed previous culturable observations regarding the intra-oral distribution of particular species but also found novel taxa which have not previously been identified amongst cultured bacteria and phyla for which only limited cultivated isolates are extant.

Here we have applied a metatranscriptomic approach to study 3 predominant oral *Veillonella* spp. in their natural habitat and have shown that their gene expression profiles are overall similar in both caries lesions and saliva (caries-free) samples. However, through differential expression analysis, *V. parvula* seems to exhibit a distinct method of intra-cellular pH control not evident in the other two species investigated, which might explain the preponderance of *V. parvula* in carious lesions and the reduced ability of *V. atypica* and *V. dispar* to proliferate in this acid environment. Other important functions related to membrane transport systems are reported to be over-expressed in the caries lesions inferring a role in disease.

We have shown here that RNA-Seq is a powerful technique that can be used to observe the transcriptome of selected species or strain, provided their genome sequence data are available. The obvious drawbacks from such technique relate to the limited number of reference genomes available for reads mapping, and also to the fact that non-core genome sequences are not captured using the current methodology. Further analyses including larger samples and samples from similar biofilms such as plaque instead of saliva would be beneficial to add to our understanding of the oral microbial functions during initiation and development of disease.

### Conflict of interest statement

The authors declare that the research was conducted in the absence of any commercial or financial relationships that could be construed as a potential conflict of interest.

## Supplementary material

Supplementary File1 **Read count data for the 144 oral strains (including *V. parvula* DSM2008, *V. dispar* ATCC 17748 and *V. atypica* ACS 0049 V Sch6), used as input file for analyses using DESeq and DESeq2 in R**.

Supplementary File2 **Relative median expression (RME) values based on the median and 75th percentile (Q3) values of normalized read counts in the caries (*n* = 11) and saliva samples (*n* = 11), for the 3 *Veillonella* species**.

Supplementary File3 **Data output from the differential expression analysis for the 3 *Veillonella* species, obtained from DESeq2 analysis**.

Supplementary File4 **Heatmap constructed using the R package DESeq2, displaying the highest most variable genes across all samples (*n* = 22), the analysis was based on the 3 *Veillonella* strains only**.

Supplementary File5 **List of reference strains used in the CLC Genomics Workbench (CLC Bio, Qiagen) for RNA-Seq analysis**.

## References

[B1] Abe H. (2000). Role of histidine-related compounds as intracellular proton buffering constituents in vertebrate muscle. Biochemistry 65, 757–765 Available online at: http://www.protein.bio.msu.ru/biokhimiya/contents/v65/pdf/bcm_0757.pdf 10951092

[B2] Anders S. Huber W. (2010). Differential expression analysis for sequence count data. Genome Biol. 11:R106. 10.1186/gb-2010-11-10-r106 20979621 PMC3218662

[B3] Arif N. Do T. Byun R. Sheehy E. Clark D. Gilbert S. C. (2008). *Veillonella* rogosae sp. nov., an anaerobic, Gram-negative coccus isolated from dental plaque. Int. J. Syst. Evol. Microbiol. 58, 581–584. 10.1099/ijs.0.65093-0 18319459 PMC2884930

[B4] Aziz R. K. Bartels D. Best A. A. Dejongh M. Disz T. Edwards R. A.. (2008). The RAST Server: rapid annotations using subsystems technology. BMC Genomics 9:75. 10.1186/1471-2164-9-75 18261238 PMC2265698

[B5] Beighton D. Clark D. Hanakuka B. Gilbert S. Do T. (2008). The predominant cultivable *Veillonella* spp. of the tongue of healthy adults identified using rpoB sequencing. Oral Microbiol. Immunol. 23, 344–347. 10.1111/j.1399-302X.2007.00424.x 18582335

[B6] Belda-Ferre P. Alcaraz L. D. Cabrera-Rubio R. Romero H. Simón-Soro A. Pignatelli M.. (2012). The oral metagenome in health and disease. ISME J. 6, 46–56. 10.1038/ismej.2011.85 21716308 PMC3246241

[B7] Benítez-Páez A. Belda-Ferre P. Simón-Soro A. Mira A. (2014). Microbiota diversity and gene expression dynamics in human oral biofilms. BMC Genomics 15:311. 10.1186/1471-2164-15-311 24767457 PMC4234424

[B8] Benjamini Y. Hochberg Y. (1995). Controlling the false discovery rate: a practical and powerful approach to multiple testing. J. R. Stat. Soc. 57, 289–300.

[B9] Broadbent J. R. Larsen R. L. Deibel V. Steele J. L. (2010). Physiological and transcriptional response of Lactobacillus casei ATCC 334 to acid stress. J. Bacteriol. 192, 2445–2458. 10.1128/JB.01618-09 20207759 PMC2863488

[B10] Brockmann-Gretza O. Kalinowski J. (2006). Global gene expression during stringent response in Corynebacterium glutamicum in presence and absence of the rel gene encoding (p)ppGpp synthase. BMC Genomics 7:230. 10.1186/1471-2164-7-230 16961923 PMC1578569

[B11] Buckel W. (2001). Sodium ion-translocating decarboxylases. Biochim. Biophys. Acta 1505, 15–27. 10.1016/S0005-2728(00)00273-5 11248185

[B12] Byun R. Carlier J. P. Jacques N. A. Marchandin H. Hunter N. (2007). *Veillonella* denticariosi sp. nov., isolated from human carious dentine. Int. J. Syst. Evol. Microbiol. 57, 2844–2848. 10.1099/ijs.0.65096-0 18048736

[B13] Csonka L. N. Epstein W. (1996). Osmoregulation, in Escherichia coli and Salmonella: Cellular and Molecular Biology, eds Neidhardt F. Curtis R. III Ingraham J. Lin E. Low K. Magasanik S. E. A. (Washington DC: American Society for Microbiology Press), 1210–1223.

[B14] Delwiche E. A. Pestka J. J. Tortorello M. L. (1985). The *veillonellae:* gram-negative cocci with a unique physiology. Annu. Rev. Microbiol. 39, 175–193. 10.1146/annurev.mi.39.100185.001135 3904599

[B15] Duran-Pinedo A. E. Chen T. Teles R. Starr J. R. Wang X. Krishnan K.. (2014). Community-wide transcriptome of the oral microbiome in subjects with and without periodontitis. ISME J. 8, 1659–1672. 10.1038/ismej.2014.23 24599074 PMC4817619

[B16] Foster J. W. (1995). Low pH adaptation and the acid tolerance response of Salmonella typhimurium. Crit. Rev. Microbiol. 21, 215–237. 10.3109/10408419509113541 8688153

[B17] Hojo S. Komatsu M. Okuda R. Takahashi N. Yamada T. (1994). Acid profiles and pH of carious dentin in active and arrested lesions. J. Dent. Res. 73, 1853–1857. 7814758 10.1177/00220345940730121001

[B18] Jorth P. Turner K. H. Gumus P. Nizam N. Buduneli N. Whiteley M. (2014). Metatranscriptomics of the human oral microbiome during health and disease. MBio 5, e01012–e01014. 10.1128/mBio.01012-14 24692635 PMC3977359

[B19] Jumas-Bilak E. Carlier J. P. Jean-Pierre H. Teyssier C. Gay B. Campos J.. (2004). *Veillonella* montpellierensis sp. nov., a novel, anaerobic, Gram-negative coccus isolated from human clinical samples. Int. J. Syst. Evol. Microbiol. 54, 1311–1316. 10.1099/ijs.0.02952-0 15280307

[B20] Love M. I. Huber W. Anders S. (2014). Moderated estimation of fold change and dispersion for RNA-seq data with DESeq2. Genome Biol. 15, 550. 10.1186/s13059-014-0550-8 25516281 PMC4302049

[B21] Luo C. Tsementzi D. Kyrpides N. Read T. Konstantinidis K. T. (2012). Direct comparisons of Illumina vs. Roche 454 sequencing technologies on the same microbial community DNA sample. PLoS ONE 7:e30087. 10.1371/journal.pone.0030087 22347999 PMC3277595

[B22] Mashima I. Kamaguchi A. Nakazawa F. (2011). The distribution and frequency of oral *veillonella* spp. in the tongue biofilm of healthy young adults. Curr. Microbiol. 63, 403–407. 10.1007/s00284-011-9993-2 21850474

[B23] Mays T. D. Holdeman L. V. Moore W. E. C. Rogosa M. Johnson J. L. (1982). Taxonomy of the genus *Veillonella*. Int. J. Syst. Bacteriol. 32, 28–36.

[B24] McLean J. S. (2014). Advancements toward a systems level understanding of the human oral microbiome. Front. Cell. Infect. Microbiol. 4:98. 10.3389/fcimb.2014.00098 25120956 PMC4114298

[B25] Munson M. A. Banerjee A. Watson T. F. Wade W. G. (2004). Molecular analysis of the microflora associated with dental caries. J. Clin. Microbiol. 42, 3023–3029. 10.1128/JCM.42.7.3023-3029.2004 15243054 PMC446285

[B26] Palmer R. J. J. Diaz P. I. Kolenbrander P. E. (2006). Rapid succession within the *Veillonella* population of a developing human oral biofilm *in situ*. J. Bacteriol. 188, 4117–4124. 10.1128/JB.01958-05 16707703 PMC1482915

[B27] Peterson S. N. Meissner T. Su A. I. Snesrud E. Ong A. C. Schork N. J.. (2014). Functional expression of dental plaque microbiota. Front. Cell. Infect. Microbiol. 4:108. 10.3389/fcimb.2014.00108 25177549 PMC4132376

[B28] Pinto A. C. Melo-Barbosa H. P. Miyoshi A. Silva A. Azevedo V. (2011). Application of RNA-seq to reveal the transcript profile in bacteria. Genet. Mol. Res. 10, 1707–1718. 10.4238/vol10-3gmr1554 21863565

[B29] Preza D. Olsen I. Aas J. A. Willumsen T. Grinde B. Paster B. J. (2008). Bacterial profiles of root caries in elderly patients. J. Clin. Microbiol. 46, 2015–2021. 10.1128/JCM.02411-07 18385433 PMC2446847

[B30] Rogosa M. (1984). Anaerobic Gram-negative cocci, in Bergey's Manual of Systematic Bacteriology, Vol. 1, eds Krieg N. R. Holt J. G. (Baltimore, MD: Williams & Wilkins), 680–685.

[B31] Simon-Soro A. Tomas I. Cabrera-Rubio R. Catalan M. D. Nyvad B. Mira A. (2013). Microbial geography of the oral cavity. J. Dent. Res. 92, 616–621. 10.1177/0022034513488119 23674263

[B32] Stumpe S. Schlosser A. Schleyer M. Bakker E. (1996). K+ circulation across the prokaryotic cell membrane: K+-uptake systems. Transport processes in eukaryotic and prokaryotic organisms, in Handbook of Biological Physics, eds Konings K. Kaback H. Lolkema J. (Amsterdam: Elsevier), 473–499.

[B33] Xiao J. Klein M. I. Falsetta M. L. Lu B. Delahunty C. M. Yates J. R. III. (2012). The Exopolysaccharide Matrix Modulates the Interaction between 3D Architecture and Virulence of a Mixed-Species Oral Biofilm. PLoS Pathog. 8:e1002623. 10.1371/journal.ppat.1002623 22496649 PMC3320608

[B34] Zeng L. Choi S. C. Danko C. G. Siepel A. Stanhope M. J. Burne R. A. (2013). Gene regulation by CcpA and catabolite repression explored by RNA-Seq in Streptococcus mutans. PLoS ONE 8:e60465. 10.1371/journal.pone.0060465 23555977 PMC3610829

