# Supplementary material for: Transcriptomic analysis of three Veillonella spp. present in carious dentine and in the saliva of caries-free individuals
Source: Front Cell Infect Microbiol. 2015 Mar 26;5:25. doi: 10.3389/fcimb.2015.00025 (PMC4374535; doi:10.3389/fcimb.2015.00025)
Supplement: Supplementary File1 — Read count data for the 144 oral strains (including V. parvula DSM2008, V. dispar ATCC 17748 and V. atypica ACS 0049 V Sch6), used as input file for analyses using DESeq and DESeq2 in R. [file DataSheet1.ZIP › SupplementaryFile5.docx]

| **AccessCode** | **Species** | **Strain** | **Seq IDs** |
| --- | --- | --- | --- |
| NZ_ACIN | *Abiotrophia defectiva* | ATCC 49176 |  |
| WE8B-23 | *Actinomyces oris* [1] | WE8B-23 |  |
| P6N | *Actinomyces oris* [4A] | P6N |  |
| CCUG33920 | *Actinomyces oris*[4B] | CCUG33920 |  |
| R21091 | *Actinomycesoris* [5] | R21091 |  |
| A7A-1 | *Actinomyces oris* | A7A-1 |  |
| NCTC10301 | *Actinomycesnaeslundii* | NCTC10301 |  |
| ACRN | *Actinomyces graeventzii* | C83 |  |
| ACTB | *Actinomyces johnsonii* | F0330 |  |
| ACYT | *Actinomyces odontolyticus* | F0309 |  |
| *A. oris*_MG1 | *Actinomyces oris* | MG1 |  |
| AFBL | *Actinomyces OT 170* | F0386 |  |
| AECW | *Actinomyces OT 171* | F0337 |  |
| AFUR | *Actinomyces OT175* | F0384 |  |
| AEUH | *Actinomyces OT178* | F0338 |  |
| AFQC | *Actinomyces OT448* | F0400 |  |
| ACUY | *Actinomyces OT848* | F0332 |  |
| AEPP | *Actinomyces OT180* | F0310 |  |
| NC_013416 | *Aggregatibacter actinomycetemcomitans* | D11S_1 |  |
| AEWB | *Actinobacillus aphrophilus* |  |  |
| NZ_ACFE | *Atopobium rimae* | ATCC_49626 |  |
| NC_013203 | *Atopobiun parvulum* | DSM 20469 |  |
| AAXD | *Bifidobacterium adolescentis* | L2-32 |  |
| ACCG | *Bifidobacterium breve* | DSM 20213 |  |
| NC_013714 | *Bifidobacterium dentium* | Bd1 |  |
| NC_004307 | *Bifidobacterium longum* | NCC2705 |  |
| ACHI | *Bifidobacterium longum subsp infantis* | ATCC55813 |  |
| NZ_ACYG | *Campylobacter gracilis* | RM3268 |  |
| NZ_ACFU | *Campylobacter rectus* | RM 3267 |  |
| AACQ | *Candida albicans* |  |  |
| NZ_ACLQ | *Capnocytophaga gingivalis* | ATCC 33624 |  |
| NC_013162 | *Capnocytophaga ochracea* | DSM 7271 |  |
| NZ_ABZV | *Capnocytophaga sputigena* | ATCC 33612 |  |
| NZ_ACKY | *Cardiobacterium hominis* | ATCC_15826 |  |
| NZ_ACIL | *Catonella morbi* | ATCC_51271 |  |
| NZ_ACSH | *Corynebacterium matruchotii* | ATCC 14266 |  |
| NC_013170 | *Cryptobacterium curtum* | DSM_15641 |  |
| NZ_ACIM | *Dialister invisus* | DSM_15470 |  |
| NZ_ACEA | *Eikenella corrodens* | ATCC 23834 |  |
| NZ_ACON | *Eubacterium saphenun* | ATCC 49989 |  |
| CP002390 | *Filifactor alocis* | ATCC_35896 |  |
| NZ_AFQD | *Fusobacterium nucleatum subsp. animalis* | ATCC 51191 |  |
| CM000440 | *Fusobacterium nucleatum subsp. polymorphum* | ATCC 10193 |  |
| AE009951 | *Fusobacterium nucleatum subsp. nucleatum* | ATCC 25586 |  |
| NZ_AABF | *Fusobacterium nucleatum subsp. vincentii* | ATCC 49256 |  |
| NZ_ACJY | *Fusobacterium periodonticum* | ATCC 33693 |  |
| NZ_ADGG | *Fusobacterium sp.* | 1_1_41FAA | GG770374-404 |
| NZ_ACDF | *Fusobacterium sp.* | 7_1 | GG658011-028 |
| NZ_ACDE | *Fusobacterium sp.* | 4_1_13 | GG657999-8010 |
| NZ_ACRO | *Gemella haemolysans* | M341 |  |
| NZ_ACKZ | *Granulicatella adjacens* | ATCC 49175 |  |
| NZ_ACRF | *Granulicatella elegans* | ATCC 700633 | GG703805 |
| NC_015964 | *Haemophilus parainfluenza* | T3T1 |  |
| NZ_ACJW | *Kingella oralis* | ATCC 51147 |  |
| ACHN | *Lactobacillus acidophilus* | ATCC 4796 |  |
| ADNY | *Lactobacillus amyllyticus* | DSM11664 |  |
| ACGG | *Lactobacillus brevis* | ATCC27305 |  |
| NC_008502 | *Lactobacillus casei* | ATCC 334 |  |
| ACKR | *Lactobacillus crispatus* | JV-CHN |  |
| AGBU | *Lactobacillus curvatus* | CRL705 |  |
| AGFO | *Lactobacillus delbrukii* | CNCM-I-1632 |  |
| NZ_ACGI | *Lactobacillus fermentum* | ATCC_14931 |  |
| ACGI | *Lactobacillus fermentum* | ATCC 14931 |  |
| NC_008530 | *Lactobacillus gasseri* | ATCC33323 |  |
| AICN | *Lactobacillus gastricus* | PS3 |  |
| ABWG | *Lactobacillus jensenii* | 1153 |  |
| ACGR | *Lactobacillus johnsonii* | ATCC33200 |  |
| AHIT | *Lactobacillus mucosae* | LM1 |  |
| ACGY | *Lactobacillus paracasei* | ATCC25302 |  |
| AGRI | *Lactobacillus plantarum* | NC8 |  |
| AAVO | *Lactobacillus reuteri* | JCM1112 |  |
| NZ_ABWJ | *Lactobacillus rhamnosus* | HN001 |  |
| AICL | *Lactobacillus salivarius* | SMXD51 |  |
| NZ_AEQP | *Lautropia mirabilis* | ATCC_51599 |  |
| NC_013192 | *Leptotrichia buccalis* | C_1013 |  |
| NZ_AFAY | *Neisseria bacilliformis* | ATCC_BAA_1200 |  |
| NZ_ADBF | *Neisseria elongata glycolytica* | ATCC_29315 |  |
| NZ_ACEN | *Neisseria flavescens* | NRL30031_H210 |  |
| NC_014752 | *Neisseria lactamica* | ST_640 |  |
| NZ_ACDX | *Neisseria mucosa* | ATCC 25996 |  |
| NZ_ACEO | *Neisseria subflava* | NG 9703 |  |
| NZ_AFIH | *Oribacterium sp.* | Oral taxon 108 |  |
| AEON | *Parascardovia denticolens* | DSM 10105 |  |
| NZ_ABEE | *Parvimonas micra* | ATCC_33270 |  |
| NZ_ADGQ | *Peptostreptococcus stomatis* | DSM 17678 |  |
| NZ_ACNN | *Porphyromonas endodontalis* | ATCC 35406 |  |
| AE015924 | *Porphyromonas gingivalis* | W83 |  |
| NZ_ADFQ | *Prevotella amnii* | CRIS 21A-A |  |
| NZ_ACKS | *Prevotella bergensis* | DSM_17361 |  |
| NZ_ADFO | *Prevotella bivia* | JCVIHMP010 |  |
| NZ_ADWO | *Prevotella bryantii* | B14 |  |
| NZ_AEPD | *Prevotella buccae* | ATCC_33574 |  |
| NZ_ADEG | *Prevotella buccalis* | ATCC_35310 |  |
| NZ_ACBX | *Prevotella copri* | DSM_18205 |  |
| NZ_AFPW | *Prevotella dentalis* | DSM_3688 |  |
| CP002589 | *Prevotella denticola* | F0289 |  |
| NZ_AEDO | *Prevotella disiens* | FB035_09AN |  |
| NZ_AEEI | *Prevotella marshii* | DSM_16973 |  |
| NC_014371 | *Prevotella melaninogenica* | ATCC 25845 CHR2 |  |
| NC_014370 | *Prevotella melaninogenica* | ATCC 25845 CHR1 |  |
| NZ_AEWX | *Prevotella multiformis* | DSM_16608 |  |
| NZ_AFPX | *Prevotella nigrescens* | ATCC_33563 |  |
| NZ_ACZS | *Prevotella oral taxon 472* | F0295 |  |
| NZ_AEPE | *Prevotella oralis* | ATCC_33269 |  |
| NZ_ACUZ | *Prevotella oris* | F0302 |  |
| NZ_AFPY | *Prevotella pallens* | ATCC_700821 |  |
| NC_014033 | *Prevotella ruminicola* | 23 |  |
| NZ_AEQO | *Prevotella salivae* | DSM_15606 |  |
| NZ_ACIJ | *Prevotella tannerae* | ATCC_51259 |  |
| NZ_ADEF | *Prevotella timonensis* | CRIS_5C_B1 |  |
| NZ_ACVA | *Prevotella veroralis* | F0319 |  |
| ACVN | *Propionobacterium OT191* | F0233 |  |
| NZ_ADFP | *Pyramidobacter piscolens* | W 5455 |  |
| NZ_ADDW | *Rothia dentocariosa* | M 567 | GL379574- |
| NZ_ACVO | *Rothia mucilaginosa* | ATCC 25296 |  |
| GG770225 | *Scardovia inopinata* | F0304 |  |
| NZ_ACKT | *Selenomonas noxia* | ATCC_43541 |  |
| NC_015437 | *Selenomonas sputigena* | ATCC 35185 |  |
| NZ_ACIP | *Shuttleworthia satelles* | DSN 14600 |  |
| NZ_AECQ | *Solobacterium moorei* | F0204 |  |
| NZ_AFIM | *Streptococcus anginosus* | SK52 |  |
| NZ_AEQR01 | *Streptococcus australis* | ATCC_700641 |  |
| NZ_AFUP | *Streptococcus constellatus* | SK1060 |  |
| NZ_AEVC | *Streptococcus cristatus* | ATCC_51100 |  |
| CP000725 | *Streptococcus gordonii* | Challis |  |
| NZ_ABJK | *Streptococcus infantarius subsp. infantarius* | ATCC_BAA_102 |  |
| NZ_AEVD | *Streptococcus infantis* | ATCC_700779 |  |
| NZ_AFXN | *Streptococcus intermedius* | F0395 |  |
| FN568063 | *Streptococcus mitis* | B6 |  |
| NZ_AFUB | *Streptococcus mitis BV2* | SK95 |  |
| AE014133 | *Streptococcus mutans* | UA159 |  |
| NZ_AEDW | *Streptococcus oralis* | ATCC35037 |  |
| NZ_AEVE | *Streptococcus parasanguinis* | ATCC_903 |  |
| NZ_AEVF | *Streptococcus peroris* | ATCC_700780 |  |
| NZ_ACLO | *Streptococcus salivarius* | SK126 |  |
| NZ_AEPO | *Streptococcus sanguinis* | ATCC_49296 |  |
| NZ_AGGU | *Streptococcus sobrinus* | TCI-345 |  |
| NZ_AEVI | *Streptococcus vestibularis* | ATCC_49124 |  |
| TFOR | *Tannerella forsythia* | ATCC 43037 |  |
| AE017226 | *Treponema denticola* | ATCC 35405 |  |
| NZ_ACYH | *Treponema vincentii* | ATCC_35580 |  |
| NZ_AEDR | *Veillonella atypica* | ACS_049_V_Sch6 |  |
| NZ_ACIK | *Veillonella dispar* | ATCC 17748 |  |
| NC_013520 | *Veillonella parvula* | DSM 2008 |  |

The genomes were downloaded from the DNA Data Bank of Japan (<ftp://ftp.ddbj.nig.ac.jp/ddbj_database/wgs/WGS_ORGANISM_LIST.html>),NCBI (<ftp://ftp.ncbi.nih.gov/genomes/Bacteria/>) and the Broad Institute (<http://www.broadinstitute.org/>).

Sequence data for strains highlighted in RED were obtained initially from the HOMD database (<http://www.homd.org/>).

Additional *Actinomyces* spp. genome sequences were obtained in the author’s laboratory.
