# Supplementary material for: Transcriptomic analysis of three Veillonella spp. present in carious dentine and in the saliva of caries-free individuals
Source: Front Cell Infect Microbiol. 2015 Mar 26;5:25. doi: 10.3389/fcimb.2015.00025 (PMC4374535; doi:10.3389/fcimb.2015.00025)
Supplement: Supplementary File1 — Read count data for the 144 oral strains (including V. parvula DSM2008, V. dispar ATCC 17748 and V. atypica ACS 0049 V Sch6), used as input file for analyses using DESeq and DESeq2 in R. [file SupplementaryFile4.PDF › SupplementaryFile4.PDF]

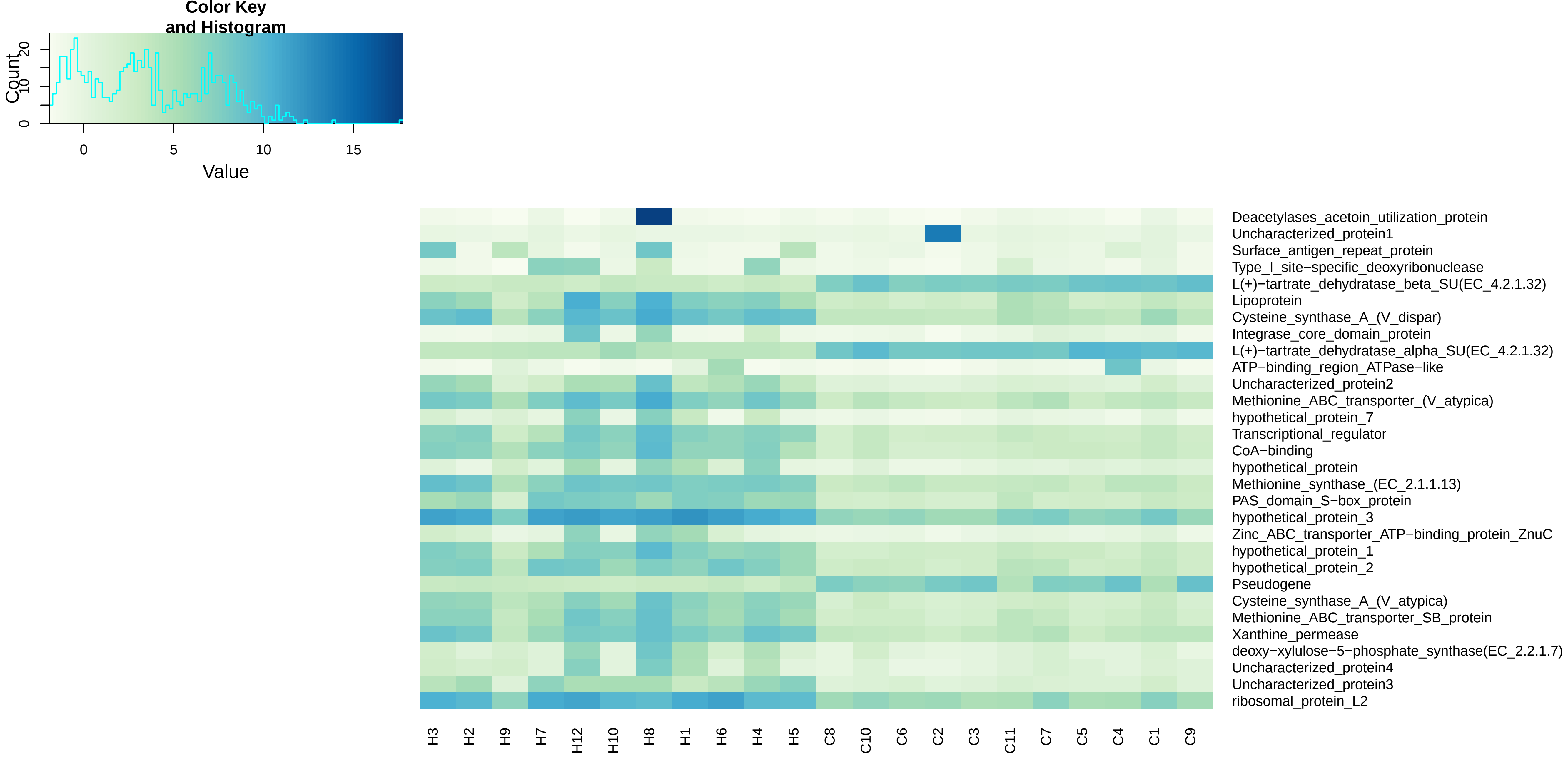
Color Key
and Histogram
Count
0
10
20
0
5
10
15
Value
Deacetylases_acetoin_utilization_protein
Uncharacterized_protein1
Surface_antigen_repeat_protein
Type_I_site−specific_deoxyribonuclease
L(+)−tartrate_dehydratase_beta_SU(EC_4.2.1.32)
Lipoprotein
Cysteine_synthase_A_(V_dispar)
Integrase_core_domain_protein
L(+)−tartrate_dehydratase_alpha_SU(EC_4.2.1.32)
ATP−binding_region_ATPase−like
Uncharacterized_protein2
Methionine_ABC_transporter_(V_atypica)
hypothetical_protein_7
Transcriptional_regulator
CoA−binding
hypothetical_protein
Methionine_synthase_(EC_2.1.1.13)
PAS_domain_S−box_protein
hypothetical_protein_3
Zinc_ABC_transporter_ATP−binding_protein_ZnuC
hypothetical_protein_1
hypothetical_protein_2
Pseudogene
Cysteine_synthase_A_(V_atypica)
Methionine_ABC_transporter_SB_protein
Xanthine_permease
deoxy−xylulose−5−phosphate_synthase(EC_2.2.1.7)
Uncharacterized_protein4
Uncharacterized_protein3
ribosomal_protein_L2
H3
H2
H9
H7
H12
H10
H8
H1
H6
H4
H5
C8
C10
C6
C2
C3
C11
C7
C5
C4
C1
C9
